# Spirulina Microalgae and Brain Health: A Scoping Review of Experimental and Clinical Evidence

**DOI:** 10.3390/md19060293

**Published:** 2021-05-22

**Authors:** Vincenzo Sorrenti, Davide Augusto Castagna, Stefano Fortinguerra, Alessandro Buriani, Giovanni Scapagnini, Donald Craig Willcox

**Affiliations:** 1 Department of Pharmaceutical and Pharmacological Sciences, University of Padova, 35131 Padova, Italy; 2 Maria Paola Belloni Center for Personalized Medicine, Data Medica Group (Synlab Limited), 35100 Padova, Italy; alessandro.buriani@gmail.com; 3 MedicRiab srls Via Novara, 6, 36071 Arzignano, Italy; dott.davidecastagna@gmail.com; 4 IRCCS SDN, 80143 Napoli, Italy; stefano.fortinguerra@gmail.com; 5 Department of Medicine and Health Sciences “V. Tiberio”, University of Molise, 86100 Campobasso, Italy; giovanni.scapagnini@unimol.it; 6 Department of Human Welfare, Okinawa International University, Ginowan 901-2701, Japan; dcwillcox@hotmail.com; 7 Department of Research, Kuakini Medical Center, Honolulu, HI 96817, USA

**Keywords:** spirulina, seaweeds, brain health, BDNF, nutraceuticals

## Abstract

Spirulina microalgae contain a plethora of nutrient and non-nutrient molecules providing brain health benefits. Numerous in vivo evidence has provided support for the brain health potential of spirulina, highlighting antioxidant, anti-inflammatory, and neuroprotective mechanisms. Preliminary clinical studies have also suggested that spirulina can help to reduce mental fatigue, protect the vascular wall of brain vessels from endothelial damage and regulate internal pressure, thus contributing to the prevention and/or mitigating of cerebrovascular conditions. Furthermore, the use of spirulina in malnourished children appears to ameliorate motor, language, and cognitive skills, suggesting a reinforcing role in developmental mechanisms. Evidence of the central effect of spirulina on appetite regulation has also been shown. This review aims to understand the applicative potential of spirulina microalgae in the prevention and mitigation of brain disorders, highlighting the nutritional value of this “superfood”, and providing the current knowledge on relevant molecular mechanisms in the brain associated with its dietary introduction.

## 1. Introduction

The brain is the body’s central control center for most physiological activities processing, integrating, and coordinating the information from peripheral sense organs, and responding with centrally elaborated instructions appropriately conveyed back to the periphery in each area of the body [1]. Keeping a healthy brain is thus fundamental to maintaining a correct function in every aspect of physiological, psychological, and social life. Following a healthy lifestyle and providing dietary nutrients and non-nutrient molecules can help to prevent or mitigate brain disorders that in turn, can affect the entire body’s functions [1,2]. Brain disorders include several conditions or disabilities that affect the brain and are caused by degenerative diseases, mental illnesses, and genetic or traumatic injuries [2]. It is known how diet can contribute to and influence the development of several neurodegenerative and mental diseases, in particular the lack of specific molecules essential for the brain [2]. Proper dietary factors can contribute to maintaining neuronal functions and synaptic plasticity, through the activation of pivotal biological mechanisms underlying brain health and mental functions. For instance, a diet rich in omega 3, choline, magnesium, B vitamins and vitamin D, specific amino acids, and phytoderivates derived from plants or seaweeds, among others, can promote brain health and preserve mental functions while reducing the onset of neurodegenerative and mental diseases [2,3].

Seaweeds provide a vast series of micro- and macronutrients (e.g., B vitamins, minerals, amino acids, etc.) and phytochemical compounds relevant for brain health. When included in the diet, they first interact with the microbiome residing in the gastrointestinal tract [4,5]. Seaweed–microbiota interaction can lead to the production of small bioactive molecules, which can affect intestinal ecology and subsequently host brain health by growth-promoting (prebiotic) effects of specific bacterial genera involved in the production of neurotransmitters, such as GABA and serotonin [4,6]. Experimental evidence has shown that bioactive seaweed-derivatives can reach and enter the brain and modulate multiple neuronal functions both directly, through specific neuronal molecules and antioxidant and anti-inflammatory activity, and indirectly by epigenetic mechanisms affecting the transcription of proteins involved in neurotransmissions, neuronal survival, and plasticity [7,8,9,10,11,12,13]. 

Studies on human subjects are increasingly associating seaweeds with neuroprotective and cognitive-enhancing actions. This includes *Ulva lactuca*, *Laminaria japonica*, *Ascophyllum nodosum*, *Fucus vesiculosus,* and *Spirulina* spp. [14,15,16]. In particular, *Spirulina* spp., given their high content of amino acids, vitamins, minerals, and a peculiar phytocomplex, are currently under investigation for their potential neuroprotective and cognitive-enhancing functions [17].

This review focuses on the beneficial effects of spirulina microalgae in the prevention or mitigation of brain disorders by analyzing the potential neuroprotective molecular mechanisms highlighted in current experimental data in vitro and in vivo studies, as well as in preliminary clinical observations that seem to encourage the use of spirulina for brain health.

## 2. Spirulina Microalgae: Sources, Chemical Composition, and Bioavailability

Spirulina (*Arthrospira platensis* (Nordstedt) Gomont, or *Spirulina platensis*) is a species belonging to the Cyanobacteria class (cyanobacteria) that lives in freshwater lakes with alkaline and warm waters (e.g., Lake Texcoco, Lake Chad, etc.) [11,18]. Along with *Spirulina platensis,* another commonly used species of spirulina for food supplements is *Spirulina maxima* [19,20]. Both species have a long history of use as food and can grow in many places worldwide thanks to an astonishing ability to thrive in climatic conditions that are adverse to other algae’s growth. Today, the main growing habitats of spirulina are the Pacific Ocean near Japan and Hawaii, and large freshwater lakes, including Lake Chad in Africa, Lake Klamath in North America, Lake Texcoco in Mexico, and Lake Titicaca in South America. Nowadays, spirulina’s world production as a food supplement is mainly in special algal farms in outdoor tanks and bioreactors [21]. The United States leads the world’s production, followed by Thailand, India, Japan, and China. The nutrient content varies considerably and depends on the production area, the climate, and the water’s salinity in which the algae grow. Harvesting procedures can also influence the content of vitamins, minerals, and phytoderivatives. Furthermore, production processes should always guarantee the absence of heavy metals that can quickly accumulate in the algae [18,21].

**Figure 1:**
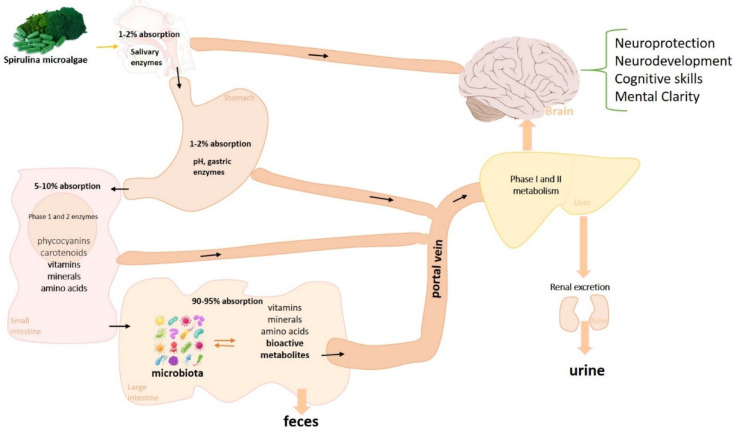
Putative absorption, metabolism, and distribution in the CNS of spirulina nutrients and phyto-derivatives. Spirulina microalgae contain a plethora of nutrient molecules and phyto-derivatives which, once taken orally, can follow different ways of absorption. In particular, most of the nutrients such as minerals, vitamins, and amino acids are rapidly absorbed through specific transporters present in the colon and duodenum although a small part can also be absorbed at the sublingual level and in the stomach. The phyto-derivatives, on the other hand, mainly undergo metabolism by phase 1 and 2 enzymes residing in the small intestine despite the majority of phytoderivates metabolism taking place in the duodenum by the intestinal microbiota that biotransforms the phyto-derivatives into small bioactive metabolites able to enter the bloodstream [31,32,33,34].

Spirulina species have a significant content of proteins, essential amino acids, vitamins, carotenoids, minerals, essential fatty acids, polysaccharides, glycolipids, etc. [22,23,24], and for this reason, they are commonly used as functional foods whose consumption benefits human health and improves physical and mental performance [18]. The WHO pointed out that spirulina is one of the most relevant superfoods on earth, and NASA uses it for space travel, thanks to the wide range of nutrients that a small amount can provide [25]. In fact, spirulina contains a high level of B vitamins, in particular vitamin B12, and minerals including iron, calcium, zinc, magnesium, manganese, and potassium [26]. In addition, some essential fatty acids, such as gamma-linolenic acid (GLA) are present. Its phytocomplex is instead rich in pigments, including chlorophyll, phycobilins such as phycocyanin, and allophycocyanin (Table 1) [22,27,28]. It is important to note that spirulina nutrients are readily absorbed by the body and quickly restore deficient nutritional status to physiological levels [27]. In particular, the high bioavailability of micronutrients allows their rapid distribution even in the nervous system. B vitamins, magnesium, and fatty acids easily reach the brain through specific carriers exerting beneficial neuronal effects [26,28]. Moreover, as for other phytoderivates [29,30], spirulina phytocomplex can also affect the brain through a first interaction with the intestinal microbiota. In fact, preliminary in vivo evidence currently shows a bidirectional interaction between spirulina and the gut microbiota. On the one hand, the microbiota can biotransform the spirulina phytocomplex into small bioactive molecules able to reach the blood and exert their beneficial functions; on the other hand, spirulina seems to modulate the microbiota diversity towards an increase in the relative abundance of protective bacteria (Figure 1) [31,32].

In particular, the influence of the intestinal microbiota on the central and autonomic nervous system can explain the neuroprotective role of spirulina. In a striking example of trans-kingdom symbiosis, gut microbiota cooperates with their human hosts to coordinate the development and function of the nervous systems through dynamic bidirectional communication along the so-called gut–brain axis [33]. Disruptions in microbial communities have been implicated in several neurological disorders and the use of probiotic and prebiotic compounds, such as those in the spirulina phytocomplex, can preserve gut microbiota homeostasis and prevent the development of brain disorders [34].

Recent in vivo studies demonstrate that oral administration of spirulina once daily for 24 consecutive days altered the diversity, structure, and composition of the colonic microbial community at the genus level, including the relative abundance of Clostridium XIVa, Desulfovibrio, Eubacterium, Barnesiella, and Bacteroides, highlighting a dose-related modulation of the intestinal microbiota and physiological states by spirulina, which can be considered as a potential source of prebiotics for beneficial health effects through interaction with the intestinal microbiota [32]. Yu et al. also demonstrated a microbiota-effect of *Spirulina platensis* on the relative amount of Proteobacteria and the Firmicutes/Bacteroidetes ratio in fecal samples from rats fed with HFD [35].

By maintaining microbial homeostasis by reducing Proteobacteria hyperproliferation, favoring short-chain fatty acids (SCFA) production and keeping an intestinal barrier integrity, spirulina allows to reduce systemic inflammatory responses which can affect the brain health. A recent study investigated the effects of different doses of phycocyanin, one of the most common pigments in spirulina, on the gut microbiota and gut barrier integrity in mice. The results highlighted an increase in the saccharolytic bacteria of the Lachnospiraceae and Ruminococcaceae families, which can produce butyric acid, and an increase in the Rikenellaceae family, which contains hydrogen-producing bacteria. Furthermore, phycocyanin treatment reduced intestinal permeability and increased intestinal barrier function [36].

The modulation of microbiota diversity is, thus, one of the potential mechanisms of action of spirulina. The impact of spirulina microalgae on the gut microbiota homeostasis should be deeply analyzed by further in vivo studies to understand its mechanism of action at the CNS.

Human studies must expand these preliminary results as the gut microbiota’s effect on the bioavailability and biotransformation of spirulina could be crucial for understanding its actions on energy metabolism, appetite regulation, and brain health.

**Table 1:** Nutrients and Phytoderivates of spirulina microalgae relevant for brain health.

**Nutrients**	**mg/g**	**Brain biological activities and molecular mechanisms**	**Ref.**
B Vitamins (B12)	0.3–0.8 (2–4 μg/g)	Energy production, synthesis of neurotransmitters and signaling molecules, DNA/RNA synthesis/repair, genomic and non-genomic methylation, cognitive functions	[23,24,26,37]
Phosphorus	3–10	energy storage, brain metabolism	[24,37]
Magnesium	1–5	Cognitive functions, enhancement of learning abilities, working memory, and short- and long-term memory; GABA synthesis;	[24,38]
Manganese	2–4	Superoxide dismutase cofactor, influence synaptic neurotransmission,	[24,39]
BCAAs	100–200	Reduced mental fatigue, neurotransmitter synthesis, protein synthesis, food intake regulation	[40]
other amino acids	300–600	Glycine, Serotonin, Dopamine, and Glutammate/GABA synthesis	[41]
GLA	10–20	Anti-inflammatory	[42,43]
**Phytoderivates**	**mg/g**	**Brain biological activities and molecular** **mechanisms**	**Ref.**
Carotenoids (Beta-carotene, Zeaxanthin)	5–20	Neuroprotection, epigenetic regulation, improved signaling efficacy, retina protection,	[24,44,45]
Total Phycocyanins (C-Phycocyanin)	400–600 (100–300)	Neuroprotection, antioxidant, anti-inflammatory	[24,46,47]
**Others**			
Superoxide Dismutase	1080 units	Radical scavenging, neuronal protection	[44,48]

## 3. Spirulina and Brain Health: In Vivo Evidence and Related Mechanism of Action

Many in vivo studies on different animal models using spirulina have highlighted several neuroprotective effects in different brain areas. Table 2 shows the main in vivo studies that have associated the use of spirulina with a neuroprotective effect. Experimental models have been used to test neuroprotection in various circumstances, from neuroinflammation to neurodegeneration, neurotoxicity, and more. One of the earliest pieces of evidence of neuroprotection in vivo was obtained in a neuroinflammation model. In particular, spirulina administration was found to reduce the acute systemic inflammatory insult of lipopolysaccharide (LPS) in young rats, which led to a decline in neural stem cell proliferation. Indeed, a spirulina dietary supplementation was found to significantly protect the proliferative potential of hippocampal neural progenitor cells [45]. The neuroprotective effect of spirulina from neuroinflammatory insults was further confirmed in an LPS-treated rat pups model where spirulina was administered to lactating mothers. In this case, LPS-induced inflammatory IL-1β was reduced, while the LPS-induced inhibition of the expression of antioxidant γglutamylcysteine ligase catalytic subunit (γGCLC), Nrf2, brain-derived neurotrophic factor (BDNF) was reversed, possibly via normalizing effects on phosphorylated AKT (pAKT) [46]. This result was further confirmed later in a similar model. Spirulina dietary supplementation to lactating mothers was confirmed to be able to protect against LPS-induced neuroinflammation in particular by reversing the decreased antioxidant defense in the brain following inflammation, an effect associated with decreased levels of phosphorylated p38 and an increase in the anti-oxidant miRNA 146a [47]. Spirulina has been shown also to exert neuroprotective effects in experimental models of neurodegenerative diseases. In a mouse model of Alzheimer’s disease, treatment with spirulina platensis water extract was suggested to prevent loss of memory by a reduction in the deposition of the amyloid β-protein in the brain, as well as by the increase in the antioxidant activity of glutathione peroxidase and the catalase activity [49]. In a different model where a cognitive disorder was induced in mice by amyloid β 1–42 icv injection, treatment with ethanol extract of spirulina maxima ameliorated cognitive impairments by inhibiting the increased phosphorylation of glycogen synthase kinase-3 and increased glutathione and activating the BDNF/phosphatidylinositol-3 kinase/serine/threonine-protein kinase signaling pathway [50]. The anti-inflammatory/anti-oxidant properties of spirulina have been also studied in Parkinson’s disease (PD) models. In a rat α-Synuclein PD model where neurodegeneration is at least partially mediated by microglial activation, spirulina administration was shown to be neuroprotective [51]. Spirulina platensis was also shown to be neuroprotective in a 6OHDA PD rat model, both in the behavioral test and in neuronal survival, an effect possibly associated with a decrease in the inflammatory enzymes iNOS and COX-2 [52]. Spirulina maxima extract was also shown to ameliorate scopolamine-induced dementia in mice, demonstrated in the Morris water maze and passive avoidance tests. The effect was associated with the increased phosphorylation of both extracellular signal-regulated kinase (p-ERK) and p-cAMP response element-binding protein (p-CREB) as well as the increase in BDNF [53]. Neuroprotective activity by spirulina in neurodegeneration has also been shown in other in vivo models and has been suggested to be mediated by an antioxidant activity associated with the increase of superoxide dismutase, catalase, and glutathione peroxidase [54]. Models of neurotoxicity have also been used to demonstrate spirulina neuroprotection. Neurotoxicity in newborn rats due to prenatal exposure to lead acetate on brain tissues could be strongly alleviated when mothers were fed with spirulina, an effect associated with the reversal of the lead-induced oxidative stress and changes in antioxidant enzyme activities in brain tissues [55]. Similarly, spirulina platensis co-administration was shown to reverse the oxidative damage induced by acute lead acetate administration to rats. An effect that was demonstrated to be mediated by the induction of caspase 3 gene expression in a similar rat model of lead-induced neurotoxicity, using spirulina maxima [56]. Spirulina platensis also demonstrated a neuroprotective activity in other neurotoxic paradigms, in particular in sodium fluoride and manganese treated animals, always associating the protective activity to an antioxidant effect [57,58]. The neuroprotective antioxidant activity of spirulina has also been demonstrated more directly in models of cerebral ischemia-reperfusion injury in rats. Pretreatment with spirulina significantly reduced the experimental neurological deficits and restored the decreased superoxide dismutase (SOD, glutathione (GSH) and catalase (CAT), indicating a clear association with antioxidant enzymatic activity [59]. Other experimental models of neuronal protection have provided further evidence of spirulina antioxidant and anti-inflammatory activity in the brain [60,61,62,63,64,65].

Taken together these data show that *Spirulina* spp. can indeed exert neuroprotection by inhibiting/reversing both inflammatory and oxidative neurotoxic mechanisms at several molecular levels in the brain.

## 4. Spirulina and Brain Health: Potential Clinical Application

Marine algae are considered a food source with unique active ingredients and potential health benefits. The antioxidant, anti-inflammatory, cholesterol homeostasis regulation, protein clearance, and anti-amyloidogenic properties confirm effective protection against oxidative stress, neuroinflammation, and mitochondrial dysfunction which are known to be implicated in the pathophysiology of neurodegenerative disorders and complications associated with cerebral ischemic events and other brain injuries. Algal compounds have been observed in various preclinical studies to confer neuroprotection against a wide range of neurotoxic stressors, such as oxygen/glucose deprivation, hydrogen peroxide, glutamate, β amyloid, or 1-methyl-4-phenylpyridinium (MPP+) and, therefore, are promising therapeutic products for brain disorders (Table 3). Numerous algal compounds with promising neuroprotective capabilities have been identified but only a few have been used in clinical trials. The approval of an algal oligosaccharide, sodium oligomannate, for the treatment of Alzheimer’s disease has recently been approved, which could guide the discovery of seaweed-based drugs [17].

In a randomized, double-blind, placebo-controlled study, the antioxidant capacity, immunomodulatory and lipid-lowering effects of spirulina administered at a dose of 8 g/day for 16 consecutive weeks in healthy elderly subjects were evaluated. In this study, a significant reduction in total plasma cholesterol levels, a significant increase in plasma interleukin IL-2 concentration and IL-6 concentration, and a significant increase in superoxide dismutase activity after supplementation were observed, demonstrating that spirulina has favorable effects on the lipid profile, on the immune system and the antioxidant capacity of elderly subjects, becoming a useful functional food [67].

Spirulina has also been shown to be able to increase people’s ability to resist mental and physical fatigue. In a randomized, double-blind, placebo-controlled study, spirulina supplementation at a dose of 3 g/day after 1 week produced a small, but statistically significant increase in exercise stamina and improved cognitive performance in as little as 4 h after the first supplementation [68]. In another randomized, double-blind clinical trial, it was observed that Arthrospira (spirulina) supplementation of 4.5 g per day, with or without an aerobic exercise program alternating with high-intensity interval training, was associated with significant reductions in all plasma lipids (decrease in total cholesterol, triglyceride, and LDL cholesterol levels and an increase in HDL cholesterol), with a decrease in the Body Mass Index [69]. In a randomized longitudinal study conducted in infants and children from Zambia, it was observed that a 16-month supplementation of spirulina improved the children’s motor development, language, and personal and social skills [70].

Beneficial effects of spirulina have also been found on blood glucose, lipids, and blood pressure levels. In a randomized, double-blind, placebo-controlled study, patients with hypertension but free from other cardiovascular diseases, after 3 months of supplementation with 2.0 g Hawaiian spirulina, showed a significant reduction in systolic blood pressure and body mass index, and an improvement of endothelial function [71]. Similar results were also obtained in another study in which *Spirulina platensis* was administered at a dose of 1 g per day for 12 weeks. This study found a decrease in total cholesterol levels and an increase in the serum concentration of HDL cholesterol, helping to control and prevent obesity-related disorders [72].

*Spirulina maxima* have shown beneficial effects not only as an antidyslipidemic but also as an antioxidant and antihypertensive. In a randomized clinical trial, it was observed that after administration of 4.5 g of *Spirulina maxima* for 12 weeks in patients with arterial hypertension there were statistically significant reductions in systolic blood pressure and levels of sVCAM-1, sE-selectin, and endothelin-1, and an increase in glutathione peroxidase activity [73].

In a double-blind, randomized, placebo-controlled clinical trial, it was found that the intake of a brown seaweed extract improved cognitive functions, in particular episodic memory and attention, in the postprandial phase and associated these effects with optimization of blood glucose levels and insulin response [16].

The identification of phytocomplexes worthy of being studied in clinical trials requires modern approaches, such as virtual screening and systems biology [74], to strengthen the development process of algae-based drugs. Computational studies could provide some crucial information on the ADME properties of potential active ingredients or phytocomplexes, and the molecular docking studies could intercept their interactions and binding affinities with molecular targets [75]. The results obtained through a systems biology approach will allow to identify potential interactions with target molecules and cell signaling pathways at the systemic level. With the constant discovery of new compounds, all of these strategies will accelerate the design and development of future algae-based drugs.

**Table 3:** Direct and indirect clinical application of spirulina microalgae on brain health.

Type of Spirulina	Subjects	Dose and Timing	Measured Parameters	Results	Reference
*Spirulina* spp.	78 healthy individuals aged 60–87 years	8 g/day for 16 weeks	Oxidative stress, inflammation and lipids-related biomarkers	Antioxidant, inflammation-lowering effect and cholesterol-lowering effect.	[67]
*Spirulina platensis*	17 healthy male individuals aged 20–43 years	3 g/day for 8 weeks	Mental and physical fatigue	Improvements in mental and physical of fatigue	[68]
*Spirulina maxima*	40 overweight and hypertensive individuals aged 40–60 years	2 g/day for 3 months	Hypertension biomarkers	Reduction in systolic blood pressure and stiffness index	[71]
*Spirulina platensis*	64 obese individuals aged 20–50 years	1 g/day for 12 weeks	Appetite	Reduction of appetite	[72]
*Spirulina maxima*	16 individuals with systemic arterial hypertension	4.5 g/day for 12 weeks	Hypertension and oxidative stress biomarkers	Reduction of systolic blood pressure and improvement in oxidative stress biomarkers	[73]
*Spirulina platensis*	501 infants aged 6–18 months	Spirulina-enriched diet, for 16 months	Motor, language and social skills development	Improvement in the measured parameters	[70]

## 5. Conclusions and Future Perspectives

Spirulina microalgae represent a source of nutrients and a phytocomplex that, taken with diet or through regulated supplementation, can support normal brain functions and the development of the nervous system, compensating for nutritional deficiencies such as those frequently encountered in developing countries. Various in vivo evidence testify to peculiar mechanisms of neuroprotection and appetite regulation, as well as antioxidant and anti-inflammatory mechanisms in the brain parenchyma with potential uses in the prevention of neurodegenerative or psychocognitive pathologies, where the inflammatory component is relevant. Preliminary clinical studies are suggesting a neuroprotective role for spirulina supplementation, especially in malnourished children, by enhancing brain development, and motor and language skills. Moreover, spirulina has been shown to improve mental and physical fatigue, probably thanks to its high levels of various brain nutrients (Table 1). Interestingly, blood pressure regulation by spirulina microalgae has been associated with stroke prevention. Despite numerous and encouraging preclinical studies, as well as some initial clinical evidence, additional clinical studies are needed to further clarify the neuroprotective actions of spirulina microalgae.

## Figures and Tables

**Table 2:** Effects of spirulina in brain health: in vivo evidence.

Type of Spirulina	Animal Model	Dose and Timing	Parameters	Results	Reference
*Spirulina* spp.	Rat (model of neuroinflammation)	Diet 0.1% *w*/*w* Spirulina for 28 days before and 2 days after LPS injection	Glial activation, neuronal progenitor cells proliferation	Protection from astrogliosis and maintainance of neuronal progenitor cells proliferation.	[45]
*Spirulina maxima*	Rat (model of ischemia-reperfusion injury)	45, 90, 180 mg/kg, for 7 days prior to middle cerebral artery occlusion	Neurological deficit, oxidative stress biomarkers, histopathological alterations in brain sections	Improvement of neurological deficit score, reduction of oxidative stress biomarkers, improvement in histopathological signs	[59]
*hSpirulina platensis*	Mouse (model of senescence/ Alzheimer’s disease)	50, 200 mg/kg/day, for 12 weeks	Memory dysfunctions, amyloid-β deposition, oxidative stress biomarkers	Improvement of the measured parameters	[49]
*Spirulina platensis*	Rat (model of lactation and lead-induced toxicity)	Diet 5% *w*/*w* Spirulina (lactating mothers), from 5th day of gestation to the 14th day of lactation, combined with lead acetate diet	Oxidative stress and histopathological alterations in brain and cerebellum in the progeny	Improvement of the measured parameters	[55]
*Spirulina* spp.	Rat (model of Parkinson’s disease)	Diet 0.1% *w*/*w* Spirulina, for 30 days before and 1, 4, 16 weeks after α-synuclein treatment	Tyrosin-Hydroxylase positive cells analysis, microglial activation	Neuroprotection, reduction of microglial activation	[51]
*Spirulina platensis*	Rat (model of pregnancy, lactation and fluoride intoxication)	250, 500 mg/kg/day, from embryonic day 6 to postnatal day 15	Neurobehavioral changes and oxidative stress in the progeny	Protection against the fluoride intoxication effects	[57]
*Spirulina maxima*	Rat (model of obesity)	1000 mg/kg/day for 30 days	Cognitive dysfunctions	Improvement of the measured parameters	[60]
*Spirulina platensis*	Rat (model of neuroinflammation)	Diet 0.1% *w*/*w* Spirulina (lactating mothers), starting 24 h before LPS injection in pups	Biochemical markers of neuroinflammation and oxidative stress	Slight improvement only for specific biomarkers	[46]
*Spirulina platensis*	Mouse (model of senescence)	400 mg/kg/day for 6 weeks	Auditory system impairments, oxidative stress biomarkers	Slight improvement only for specific auditory stimulations, reduction of oxidative stress	[54]
*Spirulina maxima*	Mouse (model of Alzheimer’s disease)	150, 450 mg/kg/day for 2 weeks before and 2 weeks after amyloid-β injection	Learning and memory dysfunctions, oxidative stress biomarkers, GSK-3β pathway	Improvement of the measured parameters, and proposal of a possible mechanism of action	[50]
*Spirulina platensis*	Rat (model of Parkinson’s disease)	25, 50 mg/kg/day for 2 weeks, starting 24 h after 6-OH-dopamine injection	Locomotor activity, biomarkers of oxidative stress and inflammation	Improvement of the measured parameters	[52]
*Spirulina maxima*	Mouse (model of dementia)	50, 100, 200, 400 mg/kg/day (no information about the duration of the treatment)	Memory dysfunctions, analysis of possibile pathways (p-ERK, p-CREB, BDNF)	Improvement of the measured parameters, and proposal of a possible mechanism of action	[53]
*Spirulina platensis*	Rat (model of lead-induced toxicity)	300 mg/kg/day for 15 days before and 15 days after lead acetate injections	Neurobehavioral alterations, oxidative stress and inflammatory response	Improvement in the behavior, and in the oxidative stress and inflammatory biomarkers	[66]
*Spirulina platensis*	Rat (lactation and neuroinflammation model)	Diet 0.1% *w*/*w* Spirulina (lactating mothers), starting 24 h before LPS injection in pups	Oxidative stress and neuroinflammation biomarkers in the progeny	Reduction of inflammation and oxidative stress in the brain	[47]
*Spirulina* spp.	Rat (model of fatigue)	300 mg/kg/day for 3 weeks	Neurotrophic signaling in hippocampal injury, and histopathological alterations in the hippocampus	Improvement of the measured parameters	[61]
*Spirulina maxima*	Rat (model of lead-induced toxicity)	500 mg/kg/day for 1 month	Oxidative stress, caspase-3 expression and histological alterations	Reduction of oxidative stress and caspase-3, improvement of histological condition of brain and cerebellum	[56]
*Spirulina platensis*	Mouse (model of photostress)	Diet 20% *w*/*w* Spirulina for 4 weeks	Visual functions, histological retinal damages, oxidative stress biomarkers	Improvement of visual functions and retinal damages, reduction of oxidative stress biomarkers	[62]
*Spirulina platensis*	Mouse (model of obesity)	2000 mg/kg/day for 4 weeks Three subtypes of Spirulina diet (whole, proteins and protein hydrolysate)	Effects on body weight, serum concentrations of lipoproteins and glucose, activation of specific pathways	Modulation of biochemical pathways in the brain–liver axis	[63]
*Spirulina platensis*	Rat (model of manganese-induced neurotoxicity	300 mg/kg for 8 weeks, alone and in combination with 50 mg/kg of alpha-lipoic acid	Neurobehavioral and biochemical changes	Detoxification from Mn and protection from the neurotoxicity	[58]
*Spirulina platensis*	Rat (model of stress)	200 mg/kg/day for 15 days, after a 2 h/10 days stress induction period	Biochemical, molecular and morphological alterations in the amygdala	Improvement of the measured parameters	[64]
*Spirulina platensis*	Rat (pregnancy, lactation and protein malnutrition model)	400 mg/kg, during gestation and lactation period	Oxidative stress, glial activation, hippocampal neuronal damage in the progeny	Protection against oxidative stress, reduction of glial activation, restoration of hippocampal cellular damage	[65]

## Data Availability

Not applicable.
